# The role of suction drainage in the management of peri-operative bleeding in Total and Unicomcompartmental knee arthroplasty: a retrospective comparative study

**DOI:** 10.1186/s12891-021-04868-4

**Published:** 2021-12-10

**Authors:** Nicola Manta, Laura Mangiavini, Cristiana Balbino, Andrea Colombo, Elisa Giacomina Pandini, Pierluigi Pironti, Marco Viganò, Roberto D’Anchise

**Affiliations:** 1 IRCCS Istituto Ortopedico Galeazzi, Milan, Italy; 2 Department of Biomedical Sciences for Health, University of Milan, Milan, Italy; 3 Residency Program in Orthopedics and Traumatology, University of Milan, Milan, Italy

**Keywords:** Total knee arthroplasty, Unicompartmental knee arthroplasty, Suction drainage, Blood loss

## Abstract

**Background:**

Suction drainage is commonly applied after total knee arthroplasty (TKA) and unicompartmental knee arthroplasty (UKA) to reduce hematoma, swelling and to favor surgical wound healing. However, its efficacy remains controversial; thus, the purpose of this study is to evaluate drainage efficiency in the management of postoperative bleeding in TKA and UKA.

**Methods:**

The cohort comprised 134 clinical records of patients affected by knee osteoarthritis (OA) who underwent either TKA or UKA. All the patients were subdivided into 2 groups: the first one with drainage and the second one without drainage (respectively 61 and 73 patients). For each group, hemoglobin levels in the preoperative, first, second and third postoperative day were collected. Postoperative complications such as swelling, bleeding from the surgical wound or the need for blood transfusion, were also recorded.

**Results:**

Our results did not show any significant difference of hemoglobin levels in the first (*p =* 0.715), second (*p =* 0.203) and third post-operative day (*p =* 0.467) between the two groups. Moreover, no significant correlation between knee swelling or transfusion rate and the drainage was observed (*p =* 0.703 and *p =* 0.662 respectively). Besides, a significant correlation was found between bleeding from the surgical wound and the absence of drainage (*p =* 0.006).

**Conclusions:**

The study demonstrates how the routine use of suction drainage does not provide substantial benefits in the postoperative blood loss management after TKA or UKA.

**Trial registration:**

ClinicalTrials.gov NCT04508101, 09/08/2020, Retrospectively registered

**Level of evidence:**

III

## Background

Knee arthroplasty is the most common treatment for osteoarthritis (OA) and other joint disorders [1, 2]. Specifically, total knee arthroplasty (TKA) is commonly considered one of the most effective orthopedic procedure in the treatment of end-stage knee OA and various rheumatic diseases such as rheumatoid arthritis, leading to substantial pain relief and functional improvement [1]. Besides, unicompartmental knee arthroplasty (UKA) represents a viable alternative in the treatment of unicompartmental knee OA showing a lower morbidity [3, 4]. Nevertheless, since knee arthroplasty involves soft tissue and bone dissection, several perioperative complications can be observed [5]. Particularly, the subsequent local bleeding may lead to anemia, functional limitation, nerve palsy and joint swelling resulting in healing impairment [1, 6]. Moreover, the local hematoma could represent the ideal environment for bacteria [6]. Although TKA usually shows a higher bleeding rate, UKAs may also have the aforementioned complications [2]. Even if it is a common belief that intra-articular suction drainage may reduce local hematoma, leading to swelling decrease, surgical wound healing improvement and reduction of postoperative infection rate [3, 4], drain use is likely to be associated with an increased risk of blood transfusion due to the lack of tamponade effect [1]. In Fast-track (FT) programs early drainage removal is recommended [4, 5]. In fact, drainage tube may interfere with physiotherapy leading to a delayed recovery [7]. Despite suction drainage is routinely used in TKA and UKA, precise guidelines are still missing and its effectiveness remains controversial [3, 8]. Thus, the aim of this study was to investigate the real usefulness of suction drainage in postoperative bleeding control in TKA and UKA.

## Methods

This retrospective, single center study was conducted at Istituto Ortopedico Galeazzi, Milan, Italy. Approval by the Ethics Committee of Ospedale San Raffaele, Milan, Italy (CE: 139/INT/2020, Milan, Italy 15/07/2020) was obtained on July 2020 and the study was registered in the ClinicalTrials.gov Registry. We analyzed 134 clinical records of patients who underwent either TKA or UKA in the period between March 2019 and March 2020. Patients were subdivided into 2 groups: the first one with drainage (D) and the second one without drainage (ND) (respectively 61 and 73 patients). All the patients underwent physical examination before surgery. Plain radiographs were performed to confirm the diagnosis and to assess OA grade. In case of clinical suspect of unicompartmental OA, magnetic resonance imaging (MRI) was prescribed to confirm the diagnosis and to assess the integrity of anterior and posterior cruciate ligament and lateral meniscus to ensure a correct treatment choice. We included patients affected by OA with either TKA or UKA indication. Patients showing contraindications to tranexamic acid (TXA), such as allergy/hypersensitivity, intrinsic risk for thrombosis or thromboembolism and hereditary thrombophilia [9] were excluded from this study. Patients undergoing revision arthroplasty were also excluded. Standard surgical procedures were applied in both groups. In the D group, an intraarticular plus a subcutaneous suction drainage were positioned for up to 24 h. Additionally, TXA was administrated to all the patients. We observed hemoglobin values before surgery and in the first, second and third postoperative day to compare the blood loss between the two groups. We also evaluated the occurrence of knee swelling, surgical wound bleeding and blood transfusions during hospitalization in both groups.

### Perioperative management

Patients included in the present study followed FT pathway. The surgery was performed by the same surgical team, specialized in knee surgery, in regional anesthesia. The patients were placed in supine position with 90 degrees’ knee flexion. In patients undergoing UKA a tourniquet was positioned at the proximal extremity of the thigh, whereas tourniquet was not applied in TKA patients. Among the 134 prostheses, a posterior stabilizing prosthesis was placed in 90 patients and a medial unicompartmental prosthesis was placed in 44 patients. For all the patients, we performed a midline skin incision and medial parapatellar approach. The prostheses were cemented following the conventional technique, using antibiotic-treated cement. Two 500 mg/5 ml TXA vials were administered systemically, and one vial locally via drainage conduct or through intraarticular injection where the drainage was not present. After surgery, standard deep vein thrombosis prophylaxis was administered for 45 days using nadroparin calcium 0,4 ml or 0,6 ml by weight (respectively < 80 kg and > 80 kg). A strict pain control was performed using analgesics and anti-inflammatory drugs to ease an early mobilization according to FT pathway. All patients underwent a rehabilitation program, which included early active and passive knee mobilization, and isometric muscle reinforcement, aiming to a faster recovery and a consequent reduction in hospitalization [10]. Patients without drainage started knee mobilization as soon as the effect of anesthesia ended; whereas, patients with drainage started knee mobilization after drainage removal. All the patients were able to walk without crutches at 45 days after surgery, and complete return to daily life activities was achieved after 3 months.

### Statistical analysis

The statistical analysis has been performed using R software v3.6.1 (R Core team, Wien, Austria). Shapiro Wilk test was used to assess the normal distribution of continuous variables. According to data distribution, parametric (unpaired Student’s t test) or non-parametric test (Wilcoxon’s test) were used to evaluate differences among the study groups. Specifically, the average of hemoglobin values and *p*-values were calculated at each time interval (t0 = pre-surgery, t1 = first day, t2 = second day, t3 = third day after surgery) for each group. Fisher’s exact test was applied to evaluate differences in proportions of categorical variables (knee swelling, wound bleeding and blood transfusions) between study groups. Moreover, regression analysis and linear models were adopted to assess the influence of more than two variables on the same parameter. Additionally, we also performed the abovementioned tests considering patients undergoing TKA and UKA separately. Statistical significance was set at *p* < 0.05.

## Results

Both groups were similar and uniform in term of sex, affected side and surgical procedure. Specifically, among 134 patients (90 TKA and 44 UKA), we performed 45 TKA and 17 UKA in D group, and 45 TKA and 27 UKA in ND group. Patients’ characteristics are summarized in Table 1. Concerning hemoglobin levels in the whole sample, we reported no statistically significant difference between the two groups at t (*p* = 0.266), t1 (*p* = 0.715), t2 (*p* = 0.203) and t3 (*p* = 0.467), showing a similar downward trend at each time interval (Table 2). Using a linear regression analysis to predict the hemoglobin loss at t3 (g/dl at t0 – g/dl at t3) considering the variables “drainage”, “sex”, “age” and “arthroplasty” (TKA or UKA), we observed that patients who underwent TKA and males tended to show a significant hemoglobin reduction (respectively *p* < 0.001 and *p = 0.038*). Moreover, there was no significant difference in knee swelling average between ND (3.6%) and D (5.6%) cases, as well as in transfusion rate (14.5 and 17.1% respectively). Indeed, double-cross tables showed no association between the presence of drainage and knee swelling (*p* = 0.703), or transfusion rate (*p* = 0.662). Furthermore, we noticed that wound bleeding was mainly present in the group without drainage (13.2% vs 1.4%), showing a significant association (*p* = 0.006) (Table 3). Nevertheless, considering “drainage”, “sex”, “age”, “hemoglobin at t0” and “arthroplasty” variables in a logistic regression model, wound bleeding showed association with the presence of drainage only (*p* = 0.020). Moreover, transfusion rates were significantly more frequent in TKA (21.4% vs 4%, *p* = 0.004) and this was confirmed (at a lower level of significance, *p* = 0.012) by a logistic regression analysis considering the same variables reported above, where also “sex” (*p* = 0.010) and the initial hemoglobin values (*p* < 0.001) demonstrated significant influence on these events. The influence of patient gender on transfusion probability was not observed in the univariate analysis (males: 21.6%; females: 13.8%; *p* = 0.300). Concerning knee swelling, we found no association neither with drainage (*p* = 0.795) nor with the other variables.

**Table 1:** Patient’s characteristics

		*Males*	*Females*	*Mean Age (SD)*
**TKA**	**Without drainage**	10	35	73.32 (6.085)
	**Drainage Group**	10	35	70.27 (9.027)
**UKA**	**Without drainage**	3	24	72.32 (8.027)
	**Drainage Group**	8	9	71.84 (8.852)

**Table 2:** Table showing mean Hb values pre-surgery as well as at 1st, 2nd and 3rd day after surgery

		*t0 (pre-surgery)*	*t1 (1st day)*	*t2 (2nd day)*	*t3 (3rd day)*
**TKA and UKA**	**Without drainage**	61	61	61	61
	**Mean Hb (SD)**	13.263 (1.413)	11.242 (1.3896)	10.543 (1.5570)	10.245 (1.4616)
	**Drainage Group**	73	73	73	73
	**Mean Hb (SD)**	13.667 (1.2612)	11.315 (1.3533)	10.473 (1.3792)	10.133 (1.4587)
	***P*-value**	0.266	0.715	0.203	0.467
**TKA**	**Without drainage**	45	45	45	45
	**Mean Hb (SD)**	13.147 (1.5135)	10.837 (1.4237)	9.970 (1.5073)	9.685 (1.3501)
	**Drainage Group**	45	45	45	45
	**Mean Hb (SD)**	13.653 (1.4263)	10.970 (1.2374)	10.063 (1.1764)	9.621 (1.1813)
	***P*-value**	0.287	0.592	0.888	0.669
**UKA**	**Without drainage**	27	27	27	27
	**Mean Hb (SD)**	13.495 (1.1905)	12.050 (0.8965)	11.690 (0.8879)	11.365 (0.9549)
	**Drainage Group**	17	17	17	17
	**Mean Hb (SD)**	13.700 (0.7237)	12.188 (1.2663)	11.512 (1.3355)	11.429 (1.3008)
	***P*-value**	0.604	0.212	0.336	0.394

Standard deviation (SD) for each value is shown in the brackets. *P*-values are given between groups pretreatment and at each follow-up

**Table 3:** Table showing the occurrence of knee swelling, need for transfusions as well as surgical wound bleeding

		*Knee swelling*	*Transfusions*	*Wound bleeding*
**TKA and UKA**	**Without drainage**	3.6%	14.5%	13.2%
	**With drainage**	5.7%	17.1%	1.4%
	***P*-value**	0.703	0.662	0.006
**TKA**	**Without drainage**	5.7%	21.2%	13.5%
	**With drainage**	5.9%	21.5%	2.0%
	***P*-value**	0.999	0.999	0.060
**UKA**	**Without drainage**	–	5.2%	12.9%
	**With drainage**	5.2%	3.2%	–
	***P*-value**	0.380	0.999	0.284

*P*-values are reported for each variable

**Fig. 1:**
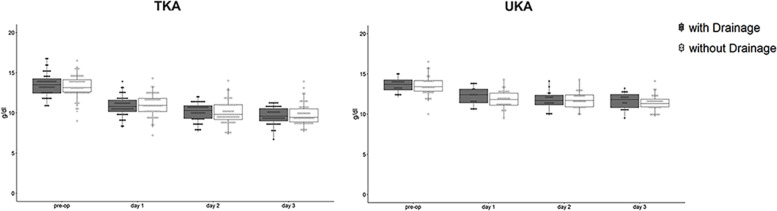
Figure showing hemoglobin trend pre-surgery as well as at 1st, 2nd and 3rd day after surgery between the two groups, among patients who underwent TKA and UKA

Considering only patients who underwent TKA, no statistically significant difference between the two groups at t0 (*p* = 0.287), t1 (*p* = 0.592), t2 (*p* = 0.888) and t3 (*p* = 0.669) was found (Table 2) (Fig. 1). The average blood loss in the drainage group among TKA treated patients was 303,60 cc. We did not observe significant difference in knee swelling between ND (5.7%) and D group (5.9%) (*p* = 0.999), and in transfusion rate (21.2 and 21.5%; *p* = 0.999). Moreover, wound bleeding was mainly reported in ND group (13.5% vs 2.0%), but this difference was not significant in the univariate analysis (*p* = 0.060) (Table 3). The same situation was observed using a logistic regression analysis considering the variables “drainage”, “sex”, “age” and “g/dl hemoglobin at t0”. This analysis also demonstrated that knee swelling is slightly more probable in males, even if in a non-significant manner (*p* = 0.075), and that the need for transfusion was higher among males (*p* = 0,020) and it was reduced with higher initial hemoglobin values (*p <* 0.001). Drainage has no influence on these events.

Patients who underwent UKA showed a comparable downward trend in hemoglobin values, showing no statistical significance at t0 (*p* = 0.604), t1 (*p* = 0.212), t2 (*p* = 0.336) and t3 (*p* = 0.394) (Table 2) (Fig. 1). The total amount of blood loss in D group among UKA treated patients was 178,68 cc. It must be noticed that knee swelling was observed only in D group (5.2%), however Fisher exact test reported no significant correlation with the lack of drainage (*p* = 0.380). The need for transfusion was instead comparable between the two groups without statistical significance (5.2% vs 3.2%, in D and ND group, respectively; *p* = 0.999). Wound bleeding was instead observed only in ND group, without statistical significance (12.9%, *p* = 0.284) (Table 3). Moreover, logistic regression analysis showed how knee swelling, transfusion rate and wound bleeding were not influenced by any of the considered variables (drainage, sex, t0 hemoglobin concentration and age).

## Discussion

In the present study emerged that drainage employment in TKA and UKA does not provide significant differences in the postoperative bleeding control. The strength of this study was in the choice to include both TKA and UKA in the analysis, considering the two procedures either as variables and separate study groups.

Knee arthroplasty is considered one of the most effective surgical procedures for OA in the orthopedic clinical practice [11]. Recently, rehabilitation protocols have been implemented to promote a faster recovery and a lower postoperative complication rate using a multimodal perioperative approach [12–14]. Particularly, pain control optimization, early mobilization and prevention of perioperative complications are required to obtain a faster recovery and a consequent shorter hospitalization [15]. It is also crucial to perform a perioperative blood management to decrease the chance of transfusions, including preoperative diagnosis, correction of anemia and an intraoperative bleeding control [9, 10]. Nowadays, several perioperative blood-preserving approaches have been described. Whereas hyperfibrinolisis resulting from surgical trauma has been proved to play a major role in perioperative bleeding, TXA administration is one of the most widely accepted methods to reduce perioperative blood loss [15–17]. Another widespread method to control intraoperative bleeding is the application of a tourniquet. Since tourniquet-related complications have been described, including wound healing impairment, thigh pain, limb swelling, nerve palsy and muscle injuries, its usefulness remains unclear [18]. Concerning suction drainage, there is still no consensus in the literature about its efficacy [3] and guidelines are not well-established, although risk and benefits of drainage use have been widely described [3, 5]. Wang et al. stated that primary TKA performed without suction drainage positioning leads to a faster recovery with an early knee function [6]. Furthermore, Zhou et al. demonstrated that tourniquet-free TKA without drainage is associated with fewer hemoglobin decrease and faster recovery [8]. In contrast, Erne et al. demonstrated an increased postoperative blood loss using suction drainage in primary TKA [19]. Nevertheless, several studies did not report significant outcome differences between a drainage and non-drainage approach [7, 20, 21]. Maniar et al. demonstrated how drainage employment, combined with TXA administration, does not influence the total blood loss and the average of swelling, range of motion, infection and deep vein thrombosis [22]. Our experience displays how suction drainage does not influence the general hemoglobin trend, knee swelling or blood transfusions needs both in TKA and UKA management (Tables 2 and 3). Even if we observed that the mean blood loss via drainage was higher in patient treated with TKA rather than UKA (Table 4), this evidence does not significantly influence hemoglobin trend (Table 2). In addition, we reported a mild delay in knee mobilization of all the patients with drainage, whose physical therapy began only after drainage removal because of patient discomfort.

**Table 4:** Table showing the mean blood loss

	*Mean blood loss (cc)*
**Total**	269.20
**TKA**	303.60
**UKA**	178.68

### Limits

Although our results are in line with the most recent medical literature, several limitations should be addressed. An extended observation would have likely provided additional information about the length of stay. However, the aim of this study was solely to assess the influence of suction drainage on postoperative blood management rather than on hospitalization length. It should be noticed that tourniquet was used only in UKA as established by our internal protocol, and so it can be assumed as part of the procedure and not as a confounding variable. Furthermore, including two different procedures could lead to bias. Nevertheless, both the studied groups were characterized by a homogeneous distribution of TKA and UKA cases, and the two procedures were also analyzed as different populations.

## Conclusions

Given our results and considering the current literature, we believe that the employment of suction drainage does not provide substantial benefits in the postoperative blood loss management in tourniquet-free TKA and in UKA performed with tourniquet.

## Data Availability

All data generated or analyzed during this study are included in this published article.

## Abbreviations

OA: Osteoarthritis
TKA: Total knee arthroplasty
UKA: Unicompartmental knee arthroplasty
FT: Fast-track
MRI: Magnetic resonance imaging
D: Drainage
ND: Non-drainage
TXA: Tranexamic acid
